# Evaluation of the Immunomodulatory Effects of Fucoidan Derived from *Cladosiphon Okamuranus* Tokida in Mice

**DOI:** 10.3390/md17100547

**Published:** 2019-09-24

**Authors:** Makoto Tomori, Takeaki Nagamine, Tomofumi Miyamoto, Masahiko Iha

**Affiliations:** 1 South Product Co., Ltd., Uruma 904-2234, Japan; miha.south@nifty.com; 2 Graduate School of Pharmaceutical Sciences, Kyushu University, Fukuoka 812-8582, Japan; miyamoto@phar.kyushu-u.ac.jp; 3 Department of Health and Nutrition, Takasaki University of Health Science, Takasaki 370-0036, Japan

**Keywords:** *Cladosiphon okamuranus*, fucoidan, immune cell proliferation, immunomodulatory

## Abstract

Okinawa mozuku (*Cladosiphon okamuranus* Tokida) is an edible seaweed classified as brown algae and is a native species of the Ryukyu Islands in Japan. In recent years, the genomic decoding of Okinawa mozuku has been completed. Previous studies on the anti-inflammatory, antiviral, and antitumor properties of Okinawa mozuku have suggested that it affects the regulation of cellular and humoral immunity. The aim of the present study was to examine the immunoregulatory effect of fucoidan derived from Okinawa mozuku in mice. A product containing fucoidan (purity, 88.3%; molecular weight, 49.8 kDa) was developed from Okinawa mozuku and tested for its immunoregulatory effects in mice. The experimental animals were 8-week-old female BALB/c mice to which fucoidan (0, 102.5, 205.0, 410.0, and 1025.0 mg/kg) was administered orally continuously for six weeks. Immune cell proliferation, cytokine production, macrophage phagocytosis, and serum antibody concentration were measured. We found that immune cell proliferation, interleukin (IL)-2, macrophage phagocytes, and serum antibodies (IgM, -G, -A) increased significantly, but IL-4, -5, and IgE decreased significantly. These results indicated that fucoidan modulated cellular and humoral immunity.

## 1. Introduction

Fucoidan is a generic term for various water-soluble sulfated polysaccharides present in brown algae. These compounds exhibit many different biological properties, including anti-inflammatory [1,2], anticoagulant [1], anti-HIV [3], and antitumor [4,5,6,7] effects. The biological properties of fucoidan vary depending on the species of algae, molecular weight, composition, and structure. Fucoidan derived from Gagome kombu (*Kjellmaniella crassifolia*) has been confirmed to be safe in healthy volunteer subjects [8] and has been reported to prevent immune function deterioration. Fucoidan derived from Mekabu (*Undaria pinnatifida*) increased helper T1 cells in BALB/c mice [9]. Okinawa mozuku (*Cladosiphon okamuranus*), the raw material of fucoidan used in this study, is an edible seaweed of the Ryukyu archipelago, Japan. The cultivation of Okinawa mozuku in Okinawa Prefecture was established by the Okinawa Prefectural Fisheries Research and Extension Center in the 1970s, and the prefecture is currently capable of providing large-scale production and a stable supply of this seaweed that accounts for >90% of domestic distribution in Japan. Recently, Nishitsuji et al. [10] decoded the draft genome of the Okinawa mozuku S-strain. This fucoidan has a linear backbone of 1–3-linked α-fucopyranose with 50% sulfate substitution at the 4-positions, and some of the fucose residues are O-acetylated [11]. This particular fucoidan is characterized by a simpler structure than that of fucoidan derived from other brown algae, Gagome kombu and Mekabu, and exhibits many biological properties, including inhibition of the adhesion of *Helicobacter pylori* [12,13], improvement of functional dyspepsia [14,15], anti-fatigue activity [6,16], and improvement in bowel movement [17,18]. A previous study on immunity reported anti-human T-cell leukemia virus type-I (HTLV-1) [19,20] and antitumor [4,5,6,7] effects. However, the mechanism underlying the immunomodulatory effects of fucoidan derived from Okinawa mozuku has not been reported. In this study, we aimed to comprehensively investigate the immunomodulatory effect of fucoidan derived from Okinawa mozuku in mice. 

## 2. Results

### 2.1. Proliferative Activity of Splenic Immune Cells

In an immune cell response, fucoidan at low (FL), middle (FM), middle high (FMH), and high (FH) doses significantly increased the proliferation activity of splenocytes stimulated by concanavalin A (Con A) and liposaccharide (LPS) (Table 1).

### 2.2. Effect of Fucoidan on Phagocytes Activity

**Figure 1:**
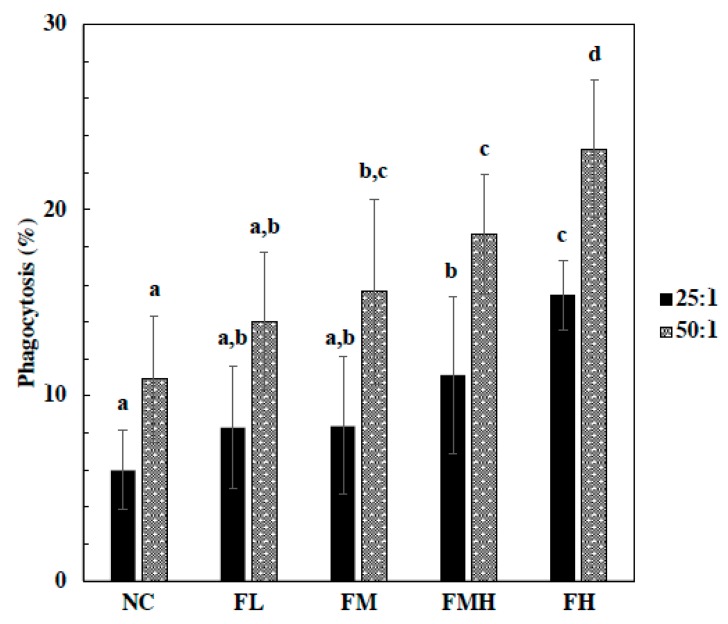
Effect of fucoidan on macrophage phagocytosis activity. The mice were administrated with fucoidan (FL, FM, FMH, FH) for 6 weeks. Peritoneal macrophage were isolated from mice and phagocytosis of fluorescence-labeled E. coli by macrophage was analyzed by flow cytometry. The macrophage to E. coli ratios were 25:1 and 50:1. All data were presented as mean ± SD *(n* = 10 mice/group). All experiments were performed in once per test condition. Different alphabets were significantly (*p* < 0.05).

In respect to phagocytes, fucoidan treatment groups at FMH and FH doses displayed significantly increased phagocytosis activity in a dose-dependent manner at an *Eschericia coli* to macrophage ratio of 25:1. Moreover, phagocytes of fucoidan groups at FM, FMH, and FH doses were significantly increased in a dose-dependent manner at an *E. coli* to macrophage ratio of 50:1 (Figure 1).

### 2.3. Effect of Fucoidan on Cytokines Production

In a cytokine production study, the splenocytes were isolated and stimulated by Con A and LPS. The levels of interleukin (IL)-2, IL-4, IL-5, and interferon (IFN)-γ were analyzed (Table 2). The levels of IL-2 in the fucoidan groups were significantly increased by Con A and LPS stimulation, but the levels of IL-4 and IL-5 were significantly decreased. The levels of IFN-γ in the fucoidan groups were significantly increased by LPS stimulation. 

### 2.4. Effect of Fucoidan on Serum Antibody Production

**Figure 2:**
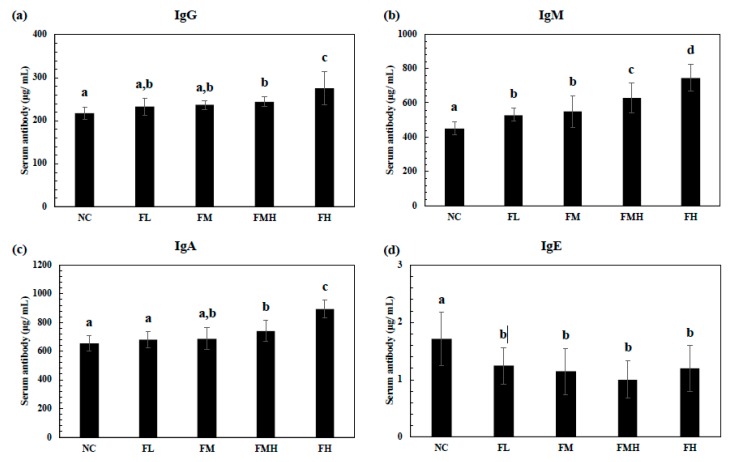
Effect of fucoidan on serum IgG (**a**), IgM (**b**), IgA (**c**), and IgE (**d**) production. The mice were administrated with fucoidan (FL, FM, FMH, FH) for 6 weeks. The serum was analyzed using ELISA assays. All data were presented as mean ± SD (*n* = 10 mice/group). All the experiments were performed in once per test condition. Different alphabets were significantly different (*p* < 0.05).

Serum immunoglobulin (Ig)G, IgM, IgA, and IgE were determined after administration of fucoidan (FL, FM, FMH, and FL) for six weeks (Figure 2). The serum antibodies in the fucoidan groups at the FMH and FH doses significantly increased in a dose-dependent manner for IgG (a) and IgA (Figure 2c). The serum IgM (Figure 2b) levels of the fucoidan groups significantly increased at all doses in a dose-dependent manner, but the serum IgE (Figure 2d) level significantly decreased.

## 3. Discussion

The bioactivity of fucoidan is affected by its chemical structure and molecular weight. In addition, the chemical structure and molecular weight affect absorption and physiological activity in the body [21,22]. Previously, it has been reported that fucoidan is involved in immune activities, such as those of macrophages, NK cells, and cytokines [23,24]. Fucoidan derived from Okinawa mozuku has been confirmed to be absorbed by rodents and humans [25,26,27]. In this study, fucoidan was shown to be active in spleen immune cells, and macrophages in mice. For cytokines, IL-2 and IFN-γ were increased, whereas IL-4 and IL-5 were reduced. Serum antibodies IgM, IgG, and IgA increased, but IgE decreased. Spleen cells and macrophage activity are affected by cytokines. The immune cells in the spleen are T and B cells. In this study, splenic immune cells were increased in the fucoidan administration groups. Con A promotes the differentiation of T cells, and LPS is a mitogen that promotes the differentiation of B cells. Fucoidan has been shown to activate the growth of T and B cells present in the spleen. Shimizu et al. [28] found that different molecular weights of fucoidan had different effects on the growth of T cells and NK cells derived from the spleen; they found that there was a greater effect on high molecular weight (2 × 10^5^ to 3 × 10^5^) fucoidan than on low molecular weight (1 × 10^3^ to 9 × 10^3^) fucoidan. The fucoidan used in this study was also high molecular weight and gave similar results. Jang et al. [29] reported that they observed a proliferative effect on mouse spleen cells by use of high molecular weight fucoidan (130 kDa). In our study, production of IL-2 and IFN-γ were increased in the fucoidan administration groups. These results suggest that macrophage phagocytes’ activity was stimulated. The activity of macrophages also has another mechanism. Doi et al. [30] showed that macrophages bind to various negatively charged molecules and reported that they were involved in biological defense mechanisms and processing mechanisms. The serum antibodies IgM, IgG, and IgA were significantly increased, whereas IgE was significantly reduced. B cells are involved in antibody production, and IL-4, IL-21, TGF-β, and IFN-γ are affected by their activity. In the case of fucoidan derived from Mekabu (*Undaria pinnatifida*) used by Takai et al. [31], a fucoidan with a molecular weight of ≥2 kDa promoted IgM, IgG, and IgA, with IgE reported to be below the detection limit. These test results suggested that a class switch of B cells caused by IFN-γ was involved in the production promotion of IgM, IgG, and IgA. On the other hand, IL-4 and IL-5, which are involved in humoral immunity, were reduced in the fucoidan administration group. These results reveal that fucoidan has the ability to adjust the balance between cellular and humoral immunity.

## 4. Materials and Methods

### 4.1. Fucoidan

In this study, we used a fucoidan product called Mei Hai Yun^®^, which was provided by Kanwa Healthcare Ltd. (New Taipei, Taiwan). Fucoidan extracted from *Cladosiphon okamuranus* Tokida (Okinawa mozuku) was manufactured by South Product Co., Ltd. (Uruma, Japan). The characteristics of this fucoidan were as follows: average molecular weight of 49.8 kDa, L-fucose content of 52.7%, uronic acid content of 18.0%, and sulfate ion content of 17.6%.

### 4.2. Animals and Treatments

Female BALB/c mice (8 weeks old) used in nonspecific study were purchased from BioLasco Taiwan Co. (Taipei, Taiwan). All animals were 8 weeks old at the start of the experiment and were housed in a normal environmentally controlled animal room (22 ± 3 °C, 12-h light/dark) with free access to pathogen-free feed and water. In this study, the mice were randomly divided into a negative control and four dose groups (102.5, 205, 410, and 1025 mg/kg body weight) with 10 mice in each group. Fucoidan was dissolved in distilled water and administered by oral gavage at 20 mL/kg body weight daily for six weeks. The negative control (0 mg/kg body weight) mice were treated identically with equal volumes of distilled water also via gavage throughout the study. All experiments were conducted according to the guidelines of the Institutional Animal Care and Use Committee of Medgaea Life Sciences.

### 4.3. Preparation of Splenocytes Suspension, Peritoneal Macrophages, and Serum

At the end of the animal experiments the mice were anesthetized and their blood was collected. The blood was centrifuged at 1200× *g* for 10 min to collect serum. The mice were injected with cold Hank’s Balanced Salt Solution (HBSS; Sigma Aldrich, St. Louis, MO, USA) into the peritoneal cavity, and the peritoneal macrophage suspension at a density of 1 × 10^6^ cell/mL was collected. The spleen was surgically removed and passed through nylon mesh (75 μm). Splenocytes were washed with RPMI1640 medium (Gibco, Grand Island, NY, USA), and red blood cells were removed by HBSS and centrifuged at 300× *g* for 10 min to collect sediment after supernatant removal. RPMI 1640 medium was added and a cell suspension was prepared at a cell density of 2 × 10^8^ cell/mL. The splenocytes suspension was used to measure proliferation and cytokine production.

### 4.4. Immune Cell Proliferation

Splenocytes were plated at a density of 4 × 10^5^ cell/mL and stimulated with concanavalin A (Con A, Sigma Aldrich, St. Louis, MO, USA) and lipopolysaccharide (LPS, Sigma Aldrich, St. Louis, MO, USA). After incubation (37 °C, 5% CO_2_, 72 h), splenocytes were stained with a CellTiter 96^®^ AQ_ueous_ One Solution Cell Proliferation Assay (Promega, Madison, WI, USA). After incubation (37 °C, 5% CO_2_, 4 h), immune cell proliferation was determined by measuring the absorption at optical density (OD) of 490 nm. Results were expressed as the stimulation index (S.I). The formula for calculating S.I is shown below:

S.I. = OD 490 nm of Con A and LPS-treated cells/OD 490 nm of untreated cells.

### 4.5. Phagocytic Activity

Peritoneal macrophages (density of 1 × 10^6^ cell/mL) and green fluorescent protein-labeled *E. coli* (Tunghai University, Taichung, Taiwan) were cultured (37 °C, 2 h) in RPMI1640 medium at E. coli/macrophage ratios of 25:1 and 50:1. The analytical buffer and trypan blue were added and analyzed by flow cytometry.

### 4.6. Cytokine Production

The splenocytes suspension (density of 4 × 10^5^ cell/mL) was treated with Con A and LPS. After incubation (37 °C, 5% CO_2_, 72 h), cell-free supernatant was collected and cytokines, including IL-4, IL-5 and IFN-γ, were determined by enzyme-linked immunosorbent assay (ELISA) (eBioscience, San Diego, CA, USA). In addition, IL-2 were determined by ELISA at 24 and 48 hours after Con A and LPS stimulation.

### 4.7. Determination of Serum Immunoglobulin

Serum was collected for the detection of antibodies (IgM, IgG, IgA, and IgE) by ELISA (Bethyl Laboratories, Montgomery, TX, USA). The level of antibodies was calculated according to the following formula:

ELISA unit (E.U.) = (ODsample-ODblank)/(ODnegative Control-ODblank).

### 4.8. Statistical Analysis

Results were presented as the mean ± standard deviation. Statistical analysis was performed by one-way ANOVA followed by Duncan’s multiple range test using SPSS 22.0 software (IBM, USA). A value of *p* < 0.05 was considered to be indicative of statistical significance.

## 5. Conclusions

We evaluated the immunomodulatory effects of fucoidan derived from *Cladosiphon okamuranus* in mice. Fucoidan was shown to stimulate immune cell proliferation, phagocytes, and cell-mediated immunity. These results suggest that fucoidan regulates natural immunity.

## Figures and Tables

**Table 1:** Effect of fucoidan on immune cell proliferation.

		Stimulation Index (S.I.)	
Group	Dose (mg/kg)	Con A (5.0 μg/mL)	LPS (10.0 μg/mL)
NC	-	2.59 ± 0.26 ^a^	2.10 ± 0.14 ^a^
FL	102.5	2.94 ± 0.29 ^b^	2.42 ± 0.15 ^b^
FM	205.0	3.16 ± 0.21 ^b,c^	2.45 ± 0.19 ^b^
FMH	410.0	3.40 ± 0.28 ^c,d^	2.74 ± 0.28 ^c^
FH	1025.0	3.54 ± 0.39 ^d^	2.82 ± 0.23 ^c^

All data are presented as mean ± SD (*n* = 10 mice/group). All experiments were performed once per test condition. NC, negative control, FL, low dose fucoidan; FM, middle dose fucoidan; FMH, middle high dose fucoidan; FH, high dose fucoidan; Con A, concanavalin A; LPS, liposaccharide. Different letters indicate significant differences (*p* < 0.05).

**Table 2:** Effect of fucoidan on cytokine production.

Group	Dose (mg/kg)	Con A (5.0 μg/ mL)	LPS (10.0 μg/ mL)
		IL-2 (pg/mL)	
NC	-	2625.1 ± 526.1 ^a^	34.1 ± 5.6 ^a^
FL	102.5	2944.2 ± 874.0 ^a,b^	38.9 ± 6.8 ^a,b^
FM	205.0	3184.5 ± 519.1 ^a,b^	39.3 ± 3.5 ^a,b^
FMH	410.0	3223.4 ± 539.6 ^b^	39.9 ± 5.6 ^a,b^
FH	1025.0	3249.2 ± 452.4 ^b^	41.9 ± 10.1 ^b^
		IL-4 (pg/mL)	
NC	-	51.9 ± 11.2 ^a^	14.3 ± 2.8 ^a^
FL	102.5	43.9 ± 13.2 ^a^	10.6 ± 0.9 ^b^
FM	205.0	41.4 ± 15.0 ^a,b^	10.7 ± 1.7 ^b^
FMH	410.0	42.1 ± 14.7 ^a,b^	10.2 ± 1.2 ^b^
FH	1025.0	31.2 ± 8.8 ^b^	9.7 ± 0.8 ^b^
		IL-5 (pg/mL)	
NC	-	121.9 ± 41.3 ^a^	10.0 ± 1.8 ^a^
FL	102.5	78.9 ± 14.5 ^b^	7.6 ± 1.0 ^b^
FM	205.0	71.6 ± 17.6 ^b^	7.4 ± 1.0 ^b^
FMH	410.0	68.4 ± 15.7 ^b^	7.1 ± 0.7 ^b^
FH	1025.0	65.8 ± 19.2 ^b^	7.0 ± 0.8 ^b^
		IFN-γ (ng/mL)	
NC	-	19.3 ± 4.8 ^a^	4.0 ± 0.9 ^a^
FL	102.5	20.4 ± 5.3 ^a^	5.1 ± 1.3 ^a,b^
FM	205.0	24.3 ± 6.8 ^a^	5.9 ± 2.4 ^b^
FMH	410.0	24.4 ± 7.7 ^a^	6.6 ± 1.9 ^b^
FH	1025.0	24.5 ± 5.2 ^a^	8.8 ± 2.8 ^c^

All data are presented as mean ± SD (*n* = 10 mice/group). All experiments were performed once per test condition. Different letters indicate significant differences (*p* < 0.05).
